# Asymmetrical Competition between *Aedes aegypti* and *Culex quinquefasciatus* (Diptera: Culicidae) Coexisting in Breeding Sites

**DOI:** 10.3390/insects8040111

**Published:** 2017-10-24

**Authors:** Juan C. Santana-Martínez, Jorge Molina, Jenny Dussán

**Affiliations:** 1 Centro de Investigaciones Microbiológicas (CIMIC), Departamento de Ciencias Biológicas, Universidad de los Andes, Carrera 1 No. 18A-10, Bogotá J-206, Colombia; jc.santana2010@uniandes.edu.co; 2 Centro de Investigaciones en Microbiología y Parasitología Tropical (CIMPAT), Departamento de Ciencias Biológicas, Universidad de los Andes, Carrera 1 No. 18A-10, Bogotá A-103, Colombia; jmolina@uniandes.edu.co

**Keywords:** *Aedes aegypti*, *Culex quinquefasciatus*, interspecific competition, breeding places, vector ecology, larval association

## Abstract

*Aedes aegypti* and *Culex quinquefasciatus* are mosquito vectors for several tropical diseases that represent a current public health problem. The ecological requirements for each species are different, however, both species show high biological adaptability, which promotes their coexistence in the same breeding sites. The purpose of this study was to assess the effect of larval association between *Ae. aegypti* and *Cx. quinquefasciatus* under different laboratory conditions of food supply and temperature, and under field simulated conditions like peridomestic containers. Our findings showed that under field simulated conditions there was no asymmetrical competition in mixed cultures with the different *Cx. quinquefasciatus/Ae. aegypti* ratios tested. However, under laboratory conditions in which different doses of food supply were evaluated, it was observed that competition between the two species takes place. Larval coexistence under food scarcity conditions (0.95 mg/larva) showed that *Ae. aegypti* had a greater adult emergence than *Cx. quinquefasciatus* and was capable of depriving *Cx. quinquefasciatus* of the food needed to complete metamorphosis. In an intermediate dose of food (1.9 mg/larva), the dry weight of *Cx. quinquefasciatus* adults decreased, and their larval development time increased when *Cx. quinquefasciatus/Ae. aegypti* ratio was low. Also, a temperature effect was assessed demonstrating that *Cx. quinquefasciatus* was more vulnerable to changes in temperature. We suggest that *Ae. aegypti* is more successful in exploiting microhabitats when food is scarce, due to its scrape active feeding habitats and fast larval development times. Therefore, in conditions of food paucity both species will compete, and *Ae. aegypti* larvae will prevail.

## 1. Introduction

*Aedes aegypti* and *Culex quinquefasciatus* are mosquito vectors for several important tropical diseases that represent a current public health problem such as Zika fever, yellow fever, chikungunya, West-Nile fever, filariasis, and encephalitis [1,2]. Furthermore, many studies have shown high biological adaptability for both species. For instance, considering *Ae. aegypti*, a previous study [3] reported multiple breeding sites including rock holes, tree holes, leaf axils, ground pools, and tires, among others, demonstrating a wide use of habitats by this species. Also, according to a study based on single nucleotide polymorphisms (SNPs) and four sequenced nuclear genes, *Ae. aegypti* has at least twelve polymorphic populations distributed all over Africa, Asia, and the Americas, thus demonstrating that this species is adaptively flexible and maintains significant genetic variation [4]. In addition, *Ae. aegypti* has been reported as an opportunistic feeder, capable of rapidly responding to environmental changes and possessing egg dormancy [4].

*Cx. quinquefasciatus* has also been reported as an opportunistic feeder that takes blood from a wide range of mammals and birds [5], and it has an olfactory memory, a mechanism that indicates the most accurate oviposition site to a gravid female mosquito. In addition, it is capable of proliferating in urban areas where blocked drains, pools collected from run-off domestic sewage, and pit latrines provide ideal larval environments, therefore indicating that it is a species tolerant to extreme values of pH, high organic material, and high salinity [6]. Furthermore, *Cx. quinquefasciatus* survives from egg to adults in temperatures ranging from 15 °C to 34 °C [7].

Both mosquito species, *Cx. quinquefasciatus* and *Ae. aegypti*, share similar patterns during their life cycles because they have aquatic as well as terrestrial stages [8]. The larvae of both are found in a variety of anthropogenic habitats, including artificial containers, storm drains, drainage ditches, and septic tanks [8,9,10,11,12,13,14]. Also, both species tend to colonize mainly urban and suburban areas in tropical countries due to the suitable climate conditions for their postembryonic development, resulting in overlapping distributions in much of the tropics [15,16]. However, current evidence showed that in spite of sharing life history traits, mosquito larvae of *Cx. quinquefasciatus* and *Ae. aegypti* differ in their feeding strategies and ecological requirements. *Ae. aegypti* have a shredding feeding mode and a predilection for temperatures above 25 °C, whereas *Cx. quinquefasciatus* have a collecting-filtering feeding mode and predilection for temperatures ranging 20–30 °C [7,17].

*Ae. aegypti* and *Cx. quinquefasciatus* have significant roles as vectors of disease agents and the larvae of both species can be found coexisting in the same breeding containers [18,19], despite the differences in ecological requirements described above. The aim of this study was to evaluate the effects of interspecific competition between the preimaginal stages of *Ae. aegypti* and *Cx. quinquefasciatus* under different laboratory conditions of food supply and temperature, and under field simulated conditions. The results of this study could provide useful insights for designing control population strategies in breeding sites, as well as providing additional knowledge about the interaction between larvae of both species under different environmental variables that can affect the development and survival rates of the larvae.

## 2. Materials and Methods

### 2.1. Larvae Collection

Field-coexisting *Ae. aegypti* and *Cx. quinquefasciatus* larvae were collected from artificial containers filled with rainwater located in suburban areas of San Joaquin municipality in La Mesa, Cundinamarca, Colombia (4°38′24.1″N, 74°31′17.9″W) during September 2016. A total of 11 containers was sampled, from which 5 were plastic barrels, 2 were cement laundries, and 4 were plastic buckets. Both species of mosquito *Ae. aegypti* and *Cx. quinquefasciatus* were present in all the containers and no other mosquito species were found.

### 2.2. Field Simulation Bioassays

**Figure 1:**
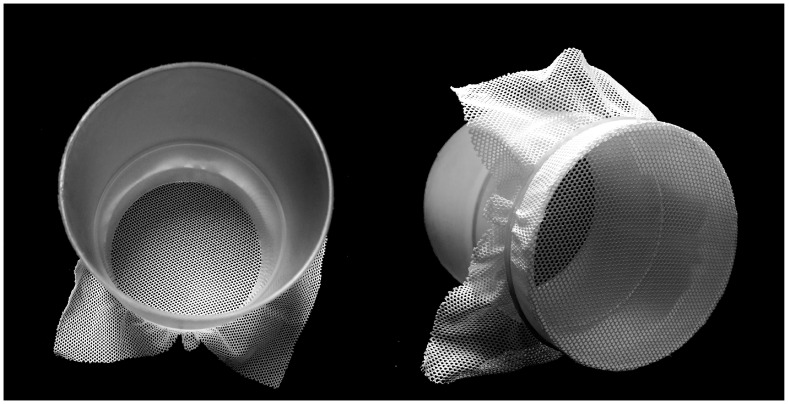
Plastic cups with a bottom net to evaluate preimaginal development and competition for food supply with different ratios between *Cx. quinquefasciatus/Ae. aegypti* under natural simulated conditions.

In order to assess the differences in preimaginal development and competition for food supply between *Ae. aegypti* and *Cx. quinquefasciatus* in natural simulated conditions of development, the following bioassays were carried out at San Joaquin: Fifteen third-instar larvae of each species were placed in 320-mL plastic cups (72 × 87 mm, width × depth) with a bottom net that allowed the water exchange (Figure 1). The following competition ratios *Cx. quinquefasciatus/Ae. aegypti*: 0:1; 1:1, and 1:0 were tested. Three 320-mL plastic cups were placed in a larger plastic container (34 × 28.5 × 11 cm, length × width × depth) filled with nine liters of rain water obtained from the same sites where the larvae were collected. The large plastic container with the small plastic cups was protected from direct sunlight.

The rate of adult production and wet body weight were registered in each experiment. To do that, the pupae obtained were separated before imaginal ecdysis, and the adults obtained were weighed (KERN 770 v2.3, KERN & SOHN GmbH ©, Balingen, Germany). The air temperature was registered in the experiment site ten times per day with intervals of approximately 2 h, and a mean of the daily data was obtained.

### 2.3. Laboratory Bioassays

Laboratory bioassays were carried out following [20]. Briefly, *Ae. aegypti* and *Cx. quinquefasciatus* larvae collected were placed in a rearing cage at 30 °C, 75% of relative humidity (RH), and light periods of 12:12 L:D. Then, adults were fed with lamb’s blood and the eggs obtained from F1 were used in our laboratory experiments. Trials were carried out in a climate-controlled chamber set at 30 °C, 75% RH, and 12:12 L:D photoperiod.

The eggs obtained from both species were placed in separate containers with dechlorinated water. Pools of first-instar larvae were separated and placed in 250 mL glass containers (previously sterilized) (Cristar ©, Medellin, Colombia) containing 200 mL of dechlorinated water. Three food doses (Omega One Super Color Cichlid Pellets, © OmegaSea LLC, Painesville, OH, USA) ground to powder and previously sterilized, were tested: 2.83; 1.9, and 0.95 mg/larva.

Additionally, the influence of air temperature was studied by comparing larval competition at 22 °C and 30 °C, using the intermediate food dose of 1.9 mg/larva, which had been previously reported as being the most appropriate dosage for evincing competition [16].

The development time, dry body weight, and rate of adult production corresponding to each temperature and food dose were quantified to establish the effect of competition with the following ratios of *Cx. quinquefasciatus/Ae. aegypti* larvae: 1:0; 2:1; 1:1; 1:2, and 0:1. Fifteen larvae were used as one unit in all the ratios (e.g., 2:1 means 30 *Cx. quinquefasciatus* larvae and 15 *Ae. aegypti* larvae).

To avoid the formation of a film on the water surface that could prevent larvae respiration, during each of the first six days of larval development the food was supplied in doses proportional to age, i.e., 10% on the first and second day, 15% on the third, 21% on the fourth, and 22% on the fifth and sixth day. Pupae were separated before imaginal ecdysis, and the adults obtained were placed at a temperature of −20 °C, dried at 80 °C for 24 h, and finally weighed (KERN 770 v2.3, © KERN & SOHN GmbH, Balingen, Germany).

### 2.4. Statistical Analysis

The software R v3.1.1 (© The R Foundation, Vienna, Austria) was used for statistical analysis [21]. In all cases normality was tested with a Shapiro–Wilk test. A Student *t*-test was carried out to evaluate the effect of competition on the rate of adult production and the wet weight of the adults obtained under field simulated conditions [22].

The development time, dry weight, and the rate of adult production obtained under laboratory conditions were analyzed by Analysis of Variance (ANOVA) followed by a Tukey-Kramer test to separate averages among the different *Cx. quinquefasciatus/Ae. aegypti* ratios tested. Among these data, few showed no normality, and they were analyzed by a Kruskal-Wallis test, followed by a Mann–Whitney *U* test to establish significant differences between treatments.

Lineal regressions with different food doses were carried out to evaluate the effect of *Cx. quinquefasciatus/Ae. aegypti* ratios on the production of adult dry biomass [22]. The competition between both species was measured using the Relative Crowding Coefficient (RCC) described by [23] and modified by [24,25]:

$$RCCs=\{\frac{\frac{1}{2}*\left(\frac{Ae^{2:1}}{Cx^{2:1}}\right)+\left(\frac{Ae^{1:1}}{Cx^{1:1}}\right)+2*\left(\frac{Ae^{1:2}}{Cx^{1:2}}\right)}{3}\}/\left(\frac{Ae^{1:0}}{Cx^{1:0}}\right)$$

When RCC > 1, competition favors *Ae. aegypti*, and vice versa, if RCC < 1, *Cx. quinquefasciatus* prevails.

## 3. Results

Data obtained in field simulation experiments showed no significant differences in the adult production rate of the two species between the three *Cx. quinquefasciatus/Ae. aegypti* ratios evaluated (Table 1). In addition, wet adult weight showed also no significant differences between the ratios tested (Table 1). Together, these results indicated that under field simulated conditions, no asymmetrical competition could be observed between both species, and coexistence was possible in the same breeding sites. Asymmetrical competition occurs when one species has a large negative effect on a competitor, which in turn has a small effect on the first species [26].

Under laboratory experiments at 30 °C, development time in *Ae. aegypti* at a food dose of 2.83 mg/larva showed no significant differences between the different *Cx. quinquefasciatus/Ae. aegypti* ratios evaluated (Table 2). However, with a food dose of 0.95 mg/larva and at ratios 2:1 and 1:1, we observed shorter times of development in *Ae. aegypti*, in contrast to those observed at ratios 0:1, 1:2, indicating a higher intraspecific vs. interspecific competition. Also, a reduction in the time of development in *Ae. aegypti* was observed with a food dose of 1.9 mg/larva at ratios of 2:1 and 1:2 compared with ratios 1:1 and 0:1 (Table 2). Although the food was supplied in mg/larva, these ratios had a higher number of larvae, suggesting that *Ae. aegypti* larvae can exploit more efficiently the resources that were initially intended for both species. Moreover, at 1.9 mg/larva (22 °C) we found significant differences between the ratios tested; however, the effect of competition and temperature on the development time at this dose remains unclear (Table 2), according to the ratios evaluated.

In the case of *Cx. quinquefasciatus*, no significant differences were found in the dose of 2.83 mg/larva (Table 2). At the dose of 0.95 mg/larva, no significant differences were found either, but in two of the tested ratios (1:1 and 1:2), no individuals of *Cx. quinquefasciatus* were obtained (Table 2). In addition, the development times obtained for *Cx. quinquefasciatus* at 1.9 mg/larva showed significant differences, with a considerable reduction in the development times in those ratios where *Ae. egypti* individuals were at minor or equal proportions (1:0, 1:1, 2:1) (Table 2). At a food dose of 1.9 mg/larva (22 °C), we found that at ratio 1:0 the development time was significantly the largest of all (Table 2). We also obtained only males at ratio 1:1 and a reduction in the time of development at the other ratios (data not shown), suggesting that the temperature is affecting more the survivorship of females than that of males. Moreover, we found that the development times were always shorter for *Ae. aegypti* than for *Cx. quinquefasciatus*, although both species were affected by the availability of resources (Table 3).

Regarding the adult production rate under laboratory conditions, *Ae. aegypti* showed no significant differences in this parameter evaluated at food dose of 1.9 mg/larva, 2.83 mg/larva, and 1.9 mg/larva (22 °C) among the different ratios tested (Table 2). At dose 0.95 mg/larva, we found significant differences between the *Cx. quinquefasciatus/Ae. aegypti* ratios, showing again a higher intraspecific vs. interspecific competition, especially at ratios 2:1 and 1:1 (Table 2).On the other hand, *Cx. quinquefasciatus,* at a food dose of 0.95 mg/larva, showed no adult production at ratios 1:2, 1:1, with more or equal individuals of *Ae. aegypti*. At a food dose of 1.9 mg/larva, we found significant differences between the ratios tested, but it seems unclear the effect of competitionon adult production at this dose. Further, at a food dose of 1.9 mg/larva (22 °C) there was a reduction in the adult production of *Cx. quinquefasciatus* at ratios 1:2, 1:1, with more or equal individuals of *Ae. aegypti*. However, with a food dose of 2.83 mg/larva no significant differences were observed in the adult production rates of *Cx. quinquefasciatus* (Table 2).

**Figure 2:**
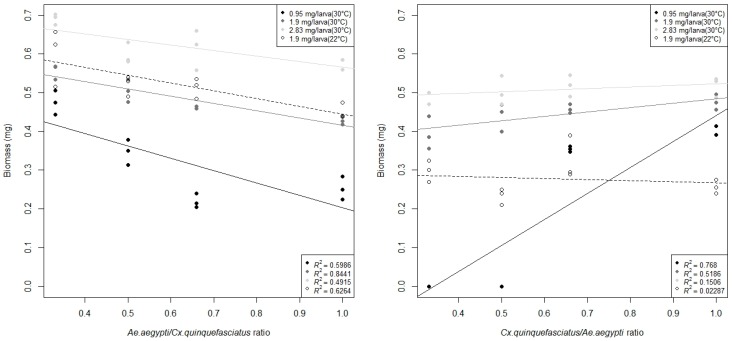
Relationship between adult dry biomass produced and different competition ratios between *Cx. quinquefasciatus/Ae. aegypti* at 22 °C or 30 °C and different food doses. *Ae. aegypti* adults (Left Panel), *Cx. quinquefasciatus* adults (Right Panel).

Considering the dry adult weight, we observed at food doses of 0.95 mg/larva, 1.9 mg/larva, and 1.9 mg/larva (22 °C) a statistical significant increase in the weight of adults of *Ae. aegypti*, directly related to the increase in *Cx. quinquefasciatus/Ae. aegypti* ratio (Table 2, Figure 2). Whereas for *Cx. quinquefasciatus,* at those same food doses, an increase in weight was obtained, inversely related to the increase in the *Cx. quinquefasciatus/Ae. aegypti* ratios (Figure 2)*.* Males at 1.9 mg/larva food dose showed no weight differences at different ratios evaluated, indicating that competition at intermediate availability of resources affected further *Ae. aegypti* and *Cx. quinquefasciatus* females. At food dose 2.83 mg/larva no correlation or significant differences were found for both species (Table 2, Figure 2), therefore implying that in cases of food shortage or under intermediate resource conditions *Ae. aegypti* biomass is negatively affected by the presence of individuals of the same species rather than by the presence of *Cx. quinquefasciatus* individuals. For *Cx. quinquefasciatus,* we found that the biomass was negatively affected by the presence of *Ae. aegypti* individuals rather than by the presence of individuals of the same species. Furthermore, the mean weight of adults at different food doses showed that although individuals of *Cx. quinquefasciatus* weigh more when there is an extensive availability of resources (2.83 mg/larva), under food scarcity there were no differences in the weight of individuals between both species, or *Ae. aegypti* tend to be heavier (Table 2). A comparison of all the biological parameters measured here showed that *Ae. aegypti* has a better efficiency in converting food supplied into biomass (Table 3).

To confirm this, our measurements using the Relative Crowding Coefficient (RCC) showed that in all food doses tested the competition between *Cx. quinquefasciatus* and *Ae. aegypti* favors the latter (RCC_0.95 (30 °C)_: 1.085547776, RCC_1.9 (30 °C)_: 1.601418242, RCC_1.9 (22 °C)_: 1.40680578), even in those cases where there was a wide availability of resources (RCC_2.83 (30 °C)_: 1.305095523). Apparently low competition was observed in food shortage (RCC_0.95 (30 °C)_: 1.085547776), which is explained by the modifications to the formula that were made due to the lack of *Cx. quinquefasciatus* individuals in the ratios 1:2 and 1:1 (Table 2).

## 4. Discussion

The main findings of this project showed that under simulated field conditions, food supply, temperature, space, and sun exposition were adequated for larval development and no asymmetrical competition was observed between both species, allowing their coexistence in the same breeding sites. However, under laboratory conditions and with different amount of food and temperatures, an asymmetrical competition was observed and *Ae. aegypti* larvae prevailed over *Cx. quinquefasciatus* larvae.

A previous study reported that at 30 °C, *Cx. quinquefasciatus* showed low dry weight gain and *Ae. aegypti* individuals showed low rates of adult production [7]. Our field-simulated conditions showed that air temperature was 30.16 ± 1.70 °C, but contrary to expectations neither adult dry weight nor rates of adult production were affected by this temperature or the co-occurrence of the two species in the same breeding site. Likewise, biological parameters previously reported as optima at this temperature (development time, weight of *Ae. aegypti,* and survival rate of *Cx. quinquefasciatus*) were also not affected, since no differences were found when the larvae were isolated from the other species.

Studies on larval development for both species [27,28,29] showed that competition for scarce food resources appeared to be more important than temperature influencing *Ae. aegypti* development in the field and in the same way more important than density influencing *Cx. quinquefasciatus* development. Field simulation bioassays were performed under shade conditions, which enhanced the overall relative productivity of pupae [30], and with 9 liters of rainwater, which enriched larvae development due to its wide content in organic matter [27]. Also, the volume of water per larva (approximately 12 mL per larva) has been reported as more than enough to avoid the density-dependent effect on *Cx. quinquefasciatus* [31,32] and *Ae. aegypti* larval development [33]. Therefore, given these adequate conditions, no competition for food or space was observed as expected.

Our experiments in field simulation conditions allow us to state that apparently under conditions of wide availability of resources, a temperature of ±30 °C, and space and sun protection, no asymmetric competition was observed. However, studies are needed to evaluate competition between the two species in locations with different temperatures, space, and protection of the sun, as well as to quantify the available food in detail and to clarify how the population dynamics fluctuate in the field. In addition, a previous study [28] showed that larval mortality might be more influenced in the early larval stages by temperature and food shortages in comparison to the last larval stages; therefore, we suggest that different early larval stages should be evaluated in further studies.

Results under laboratory conditions, at 30 °C and 22 °C, showed that *Ae. aegypti* is more successful than *Cx. quinquefasciatus* in exploiting artificial microhabitats either when food supply is scarce (0.95 mg/larva) or at food intermediate concentration (1.95 mg/larva). Thus, in conditions of food paucity, the two species were brought into competition, and *Ae. aegypti* succeeded over *Cx. quinquefasciatus*. As a consequence, an increase in the time of development, a decrease in the overall dry weight, and metamorphosis failures of *Cx. quinquefasciatus* were found (Table 2). Furthermore, RCCs measured in our experiments supported this assumption. Also, *Cx. quinquefasciatus* females seem to be more affected by food shortage, since males presented no differences in weights at intermediate conditions.

Change in temperature caused that development time of *Ae. aegypti* increase more in relation to the increase in time measured for *Cx. quinquefasciatus*. However, development time of *Ae. aegypti* at 22 °C was lower than the time for *Cx. quinquefasciatus*. As well, percentage of emergent adults and dry weight of adults of *Cx. quinquefasciatus* were affected to a greater extent by the change in temperature (Table 3). We suggest that even though *Cx. quinquefasciatus* survives from eclosion to adult emergence in a wide range of temperatures [7], only certain temperatures are most likely to reach development and to be successful. Moreover, despite the fact that *Ae. aegypti* has a narrower surviving range of temperatures, it is more efficient at converting food into biomass and having higher survival rates compared with *Cx. quinquefasciatus*. However, a recent study showed that *Ae. aegypti* is capable of reaching pupation under constant and diurnal temperatures, ranging from 25 °C to 35 °C, in 79% of the population, in the worst cases [34].

We suggest that laboratory results evinced an asymmetrical competition between both species in conditions of food paucity and apparently in a range of temperature of 22–30 °C. Intrinsic factors of each species may explain the asymmetry of this competition. (1) *Ae. aegypti* uses an active feeding mode (shredding), whereas *Cx. quinquefasciatus* is considered a passive collector-filter, which presumably leads *Ae. aegypti* to be more efficient at resource acquisition and spend more time behaviorally active foraging or feeding [17]. (2) *Ae. aegypti* larvae can feed from particulate organic matter such as leaves, filaments of macroalgae, or other plant parts, and even though dead invertebrates, often of their own kind. Instead, *Cx. quinquefasciatus* removes particulate organic material from suspension [17]. (3) Differences found in previous studies in regard to filtration rates: *Cx. quinquefasciatus* (490–590 μL/larva/h) contrastingly to *Ae. aegypti* (590–690 μL/larva/h) [35]. (4) Differences found in time of the larval development for both species; *Ae. aegypti* larvae showed faster developing times [our results and 7, 27]. (5) Resistance to starvation in third-instar *Ae. aegypti* larvae, which is capable of surviving for as long as 47 days when reared with artificial food and 24 days when reared with natural food [36]. The number of days that larvae can survive without food is a function of accumulated reserves, mainly lipids [37]; therefore, *Ae. aegypti,* due to its exponential lipogenesis [38], is capable of surviving food privation and presumably has better efficiency converting food into biomass.

Moreover, we found an interesting pattern in the dry weight of adults for both species according to the amount of individuals of the same species when food was limited. In the case of *Ae. aegypti*, we found an inverse relationship between biomass and *Ae. aegypti/Cx. quinquefasciatus* ratio, which would indicate a higher intraspecific vs. interspecific competition. On the other hand, for *Cx. quinquefasciatus* we found a direct relationship between biomass and the *Cx. quinquefasciatus/Ae. aegypti* ratio, indicating a higher interspecific vs. intraspecific competition (Figure 2). Also, the slopes of each food dose in Figure 1 demonstrated how strong the effect of intraspecific or interspecific competition was, according to the effect of food shortage. Previous studies showed similar results, indicating a positive relationship between adult size and food availability [39]. Also, studies assessing larval association between *Ae. aegypti* and *Cx. pipiens* demonstrated that *Ae. aegypti* was more affected by intraspecific competition than interspecific competition [40]. Density-dependent events in *Ae. aegypti* can be caused by mechanical interference among larvae during feeding activity. Since the encounters or collisions between individuals are greater in *Ae. aegypti*, higher intraspecific competition vs. interspecific competition might be expected [33]. Results of a laboratory-based study conclude that under interspecific competition, *Cx. quinquefasciatus* favors *Ae. aegypti* because the former reduces the amount of organic matter in the media [18]. Those findings showed the same asymmetrical competition found in our experiments; however, recent evidence [9,10,13] showed that *Ae. aegypti* occurs in containers with polluted stagnant waters rich in organic matter. Consequently, we assume intraspecific competition, and interspecific competition results might be more related to the intrinsic factors described above rather than previous findings that were related with media contamination [18].

Current evidence suggests that climate change is likely to increase the area of land with a suitable climate for *Ae. aegypti*, since increasing global temperatures and other associated climate changes may modify the mosquito’s geographic range [41,42]. Altitudes that are currently too cool to sustain vectors will become more conducive to them. Some vector populations may expand into new geographic areas, whereas others may disappear [43,44]. Therefore, according to our results and taking into account that *Ae. aegypti* is an opportunistic species which can easily colonize empty niches [4] and has the ability to adapt to higher temperature ranges [34], there seem to be no obstacles to the spread of *Ae. aegypti* deriving from competition with the species *Cx. quinquefasciatus* within a climate change scenario. Nevertheless, additional field studies are needed in this regard to elucidate how dynamics can vary if the environmental conditions change.

Similarly to previous studies suggestions [33,36] for mosquito control, it is important to consider if total elimination of all *Ae. aegypti* individuals in each container is achievable. Events lightening intraspecific competition in *Ae. aegypti* may increase body mass and consequently produce bigger adults. Larvicidals directed to mixed populations should be very efficient in order to evade the effects of alleviation of *Ae. aegypti* intraspecific competition, since *Cx. quinquefasciatus* is a weaker competitor. Concerning *Cx. quinquefasciatus*, we suggest previous assessments of the larvicidal under larval association conditions in order to avoid biocontrol overestimation and achieve more efficient formulations.

## 5. Conclusions

This is the first study to investigate larval interactions between *Ae. aegypti* and *Cx. quinquefasciatus*. In the field-simulated conditions we found that apparently with a wide availability of resources, a temperature of ±30 °C, and space and sun protection, no asymmetric competition occurs. Nevertheless, we demonstrated that *Ae. aegypti* is a superior resource competitor and appears to be capable of competitively affecting *Cx. quinquefasciatus* under conditions of limited resources and different temperatures. Although it is evidently that interspecific competition between these species is occurring and has the potential to affect vector population dynamics, there is a clear need for field investigations of both the ecological effects and epidemiological consequences of competition between these species.

## Figures and Tables

**Table 1:** Effect of competition on the biological parameters evaluated under simulated field conditions.

*Cx. quinquefasciatus/Ae. aegypti* Ratio	N	*Ae. aegypti*		*Cx. quinquefasciatus*	
		Adult Emergence (Proportion ± SD)	Wet Adult Weight (mg ± SD)	Adult Emergence (Proportion ± SD)	Wet Adult Weight (mg ± SD)
1:0	6			0.90 ± 0.06a	0.94 ± 0.03a
1:1	6	0.92 ± 0.08a	0.91 ± 0.03a	0.92 ± 0.08a	0.94 ± 0.05a
0:1	6	0.94 ± 0.08a	0.90 ± 0.05a		

Means within a column block followed by the same letter are not significantly different according to Student *t*-test.

**Table 2:** Effect of competition on the biological parameters evaluated under laboratory conditions.

*Cx. quinquefasciatus/Ae. aegypti* Ratio	N	*Ae. aegypti*			*Cx. quinquefasciatus*		
		Time from Egg to Adult (Days ± SD)	Adult Emergence (Proportion ± SD)	Dry Adult Weight (mg ± SD)	Time from Egg to Adult (Days ± SD)	Adult Emergence (Proportion ± SD)	Dry Adult Weight (mg ± SD)
0.95 mg/larva							
(30 °C)							
1:0	3				12.33 ± 0.57	0.18 ± 0.04	0.40 ± 0.01a
2:1	3	9.63 ± 0.59a	0.80 ± 0.02a	0.47 ± 0.03a	13.66 ± 0.57	0.18 ± 0.02	0.35 ± 0.01b
1:1	3	10.68 ± 0.73ab	0.78 ± 0.10a	0.35 ± 0.03b	N/A	N/A	N/A
1:2	3	12.08 ± 0.02b	0.54 ± 0.07b	0.22 ± 0.02c	N/A	N/A	N/A
0:1	3	12.27 ± 1.00b	0.24 ± 0.04c	0.25 ± 0.03c			
1.9 mg/larva							
(30 °C)							
1:0	3				8.76 ± 0.35a	0.62 ± 0.03 a	0.48 ± 0.02a
2:1	3	5.71 ± 0.64a	0.97 ± 0.04	0.56 ± 0.02a	9.23 ± 0.15a	0.93 ± 0.03b	0.46 ± 0.01ab
1:1	3	8.26 ± 0.54b	0.93 ± 0.07	0.50 ± 0.03b	8.68 ± 0.29a	0.6 ± 0.11a	0.44 ± 0.04ab
1:2	3	6.12 ± 0.23a	0.95 ± 0.08	0.46 ± 0.01bc	9.97 ± 0.25b	1.00 ± 0.00b	0.39 ± 0.04b
0:1	3	8.25 ± 0.14b	0.84 ± 0.10	0.43 ± 0.01c			
2.83 mg/larva							
(30 °C)							
1:0	3				9.19 ± 0.20	0.96 ± 0.04	0.52 ± 0.03
2:1	3	7.91 ± 0.22	0.87 ± 0.07	0.69 ± 0.01	8.93 ± 0.15	0.97 ± 0.03	0.51 ± 0.03
1:1	3	7.28 ± 0.29	0.93 ± 0.11	0.60 ± 0.03	9.29 ± 0.61	0.91 ± 0.04	0.50 ± 0.04
1:2	3	7.67 ± 0.47	0.93 ± 0.06	0.61 ± 0.05	9.00 ± 0.18	0.96 ± 0.04	0.49 ± 0.02
0:1	3	8.05 ± 0.16	0.93 ± 0.07	0.58 ± 0.01			
1.9 mg/larva							
(22 °C)							
1:0	3				17.07 ± 0.40a	0.84 ± 0.04a	0.26 ± 0.02ab
2:1	3	16.01 ± 0.55a	0.87 ± 0.00	0.60 ± 0.07a	15.43 ± 0.60b	0.74 ± 0.02a	0.32 ± 0.06b
1:1	3	13.81 ± 0.52b	0.89 ± 0.04	0.52 ± 0.03b	15.45 ± 0.51b	0.42 ± 0.08b	0.23 ± 0.02a
1:2	3	15.61 ± 0.45a	0.88 ± 0.05	0.51 ± 0.03b	14.74 ± 0.79b	0.64 ± 0.20ab	0.30 ± 0.03ab
0:1	3	15.35 ± 0.17a	1.00 ± 0.00	0.45 ± 0.02c			

Means within a column block followed by the same letter are not significantly different according to Tukey-Kramer test or Mann–Whitney *U* test in case of no normality. Student *t*-test was used in case of 2 treatments.

**Table 3:** Comparison between biological parameters (±SD) of *Ae. aegypti* and *Cx. quinquefasciatus* at different food doses under laboratory conditions.

	0.95 mg/larva (30 °C)	1.9 mg/larva (30 °C)	2.83 mg/larva (30 °C)	1.9 mg/larva (22 °C)
Proportion of adult emergence				
*Ae. aegypti*	0.59 ± 0.24a	0.92 ± 0.08a	0.92 ± 0.07a	0.91 ± 0.06a
*Cx. quinquefasciatus*	0.18 ± 0.03b	0.79 ± 0.19b	0.94 ± 0.04a	0.66 ± 0.19b
Days from egg to adult				
*Ae. aegypti*	11.17 ± 1.27a	7.10 ± 1.29a	7.73 ± 0.40a	15.20 ± 0.95a
*Cx. quinquefasciatus*	13 ± 0.89b	9.16 ± 0.59b	9.10 ± 0.33b	15.67 ± 1.03a
Mean Dry adult weight (mg)				
*Ae. aegypti*	0.32 ± 0.11a	0.49 ± 0.05a	0.51 ± 0.03a	0.56 ± 0.11a
*Cx. quinquefasciatus*	.38 ± 0.03a	0.44 ± 0.04b	0.62 ± 0.05b	0.28 ± 0.05b

Means within a column block followed by the same letter are not significantly different according to Student *t*-test.
